# The Modern Treatment of Syphilitic Diseases, Both Primary and Secondary

**Published:** 1856-01

**Authors:** 


*The Modern Treatment of Syphilitic Diseases, both Primary and Secondary.* By Langster Parker, Surgeon, &c. Third Edition. Blanchard & Lee, 1854.

This work, which we have been unable yet to give the careful perusal we design, seems in every way deserving the attention of the profession. It is systematically and well arranged, gives us a view of the subjects treated in every aspect, is illustrated by cases, registered upon a very convenient and practical method, and contains many formulæ, containing the most approved remedies of the day.

The author being surgeon to the Queen's Hospital, Birmingham, has devoted twenty years to the "Therapeutics of Syphilis," having treated more than eight thousand cases, certainly has given him every desirable advantage, and his book shows him capable of properly appreciating and clearly relating the results of his ample experience. We predict for the book a favorable reception and general reading in this country.

